# Effect of passive and active ventilation on malaria mosquito house entry and human comfort: an experimental study in rural Gambia

**DOI:** 10.1098/rsif.2022.0794

**Published:** 2023-04-05

**Authors:** Majo Carrasco-Tenezaca, Musa Jawara, Daniel Sang-Hoon Lee, Matthew S. Holmes, Sainey Ceesay, Phillip McCall, Margaret Pinder, Umberto D'Alessandro, Jakob B. Knudsen, Steve W. Lindsay, Anne L. Wilson

**Affiliations:** ^1^ Department of Biosciences, Durham University, Stockton Road, Durham, Durham DH1 3LE, UK; ^2^ Medical Research Council Unit The Gambia at the London School of Hygiene and Tropical Medicine, Fajara, The Gambia; ^3^ Architecture, Design and Conservation, The Royal Danish Academy, Philip De Langes Allé 10, Copenhagen 1435, Denmark; ^4^ JDDK Architects, Millmount, Ponteland Rd, Cowgate, Newcastle upon Tyne NE5 3AL, England; ^5^ Department of Vector Biology, Liverpool School of Tropical Medicine, Pembroke Place, Liverpool L3 5QA, UK; ^6^ London School of Hygiene & Tropical Medicine, Keppel St, Bloomsbury, London WC1E 7HT, UK

**Keywords:** malaria, ventilation, Africa, house design, human comfort, mosquito

## Abstract

Rural houses in sub-Saharan Africa are typically hot and allow malaria mosquitoes inside. We assessed whether passive or active ventilation can reduce house entry of malaria mosquitoes and cool a bedroom at night in rural Gambia. Two identical experimental houses were used: one ventilated and one unventilated (control). We evaluated the impact of (i) passive ventilation (solar chimney) and (ii) active ventilation (ceiling fan) on the number of mosquitoes collected indoors and environmental parameters (temperature, humidity, CO_2_, evaporation). Although the solar chimney did not reduce entry of *Anopheles gambiae* *sensu lato*, the ceiling fan reduced house entry by 91% compared with the control house. There were no differences in indoor nightly temperature, humidity or CO_2_ between intervention and control houses in either experiment. The solar chimney did not improve human comfort assessed using psychrometric analysis. While the ceiling fan improved human comfort pre-midnight, in the morning it was too cool compared with the control house, although this could be remedied through provision of blankets. Further improvements to the design of the solar chimney are needed. High air velocity in the ceiling fan house probably reduced mosquito house entry by preventing mosquito flight. Improved ventilation in houses may reduce malaria transmission.

## 1. Introduction

In sub-Saharan Africa, most rural houses are built from mud [1,2] and concrete, both materials with a high thermal mass, making them hot at night [2,3]. These structures are even hotter if they have metal roofs [3]. Consequently, if the room is uncomfortably hot [3,4], people may not protect themselves from malaria by sleeping under an insecticide-treated net (ITN) [5,6], as they further restrict air flow and potential cooling [7]. Considering that roughly 80% of malaria transmission in the region occurs indoors at night [8,9], low use of ITNs can increase malaria transmission. Therefore, simple and cheap methods to keep houses cool, especially at night, while keeping out malaria mosquitoes would decrease malaria transmission. Ideally this should be done without using energy expensive and greenhouse gas emitting cooling devices like air conditioners.

Ventilation, by adding at least two large screened windows in opposite walls of a single-room house is one method by which rural homes can be cooled, by replacing hot, static indoor air with cooler air flow from outdoors [10]. Improving ventilation indoors also reduces the levels of CO_2_ in a room both directly by removing the gas from the room and indirectly by reducing the room temperature and thus lowering the human metabolic activity [10]. Reducing CO_2_ concentrations indoors will make it more difficult for a mosquito to locate and feed on a person since this gas is the major long-distance attractant for malaria mosquitoes [11,12].

**Figure 1.**
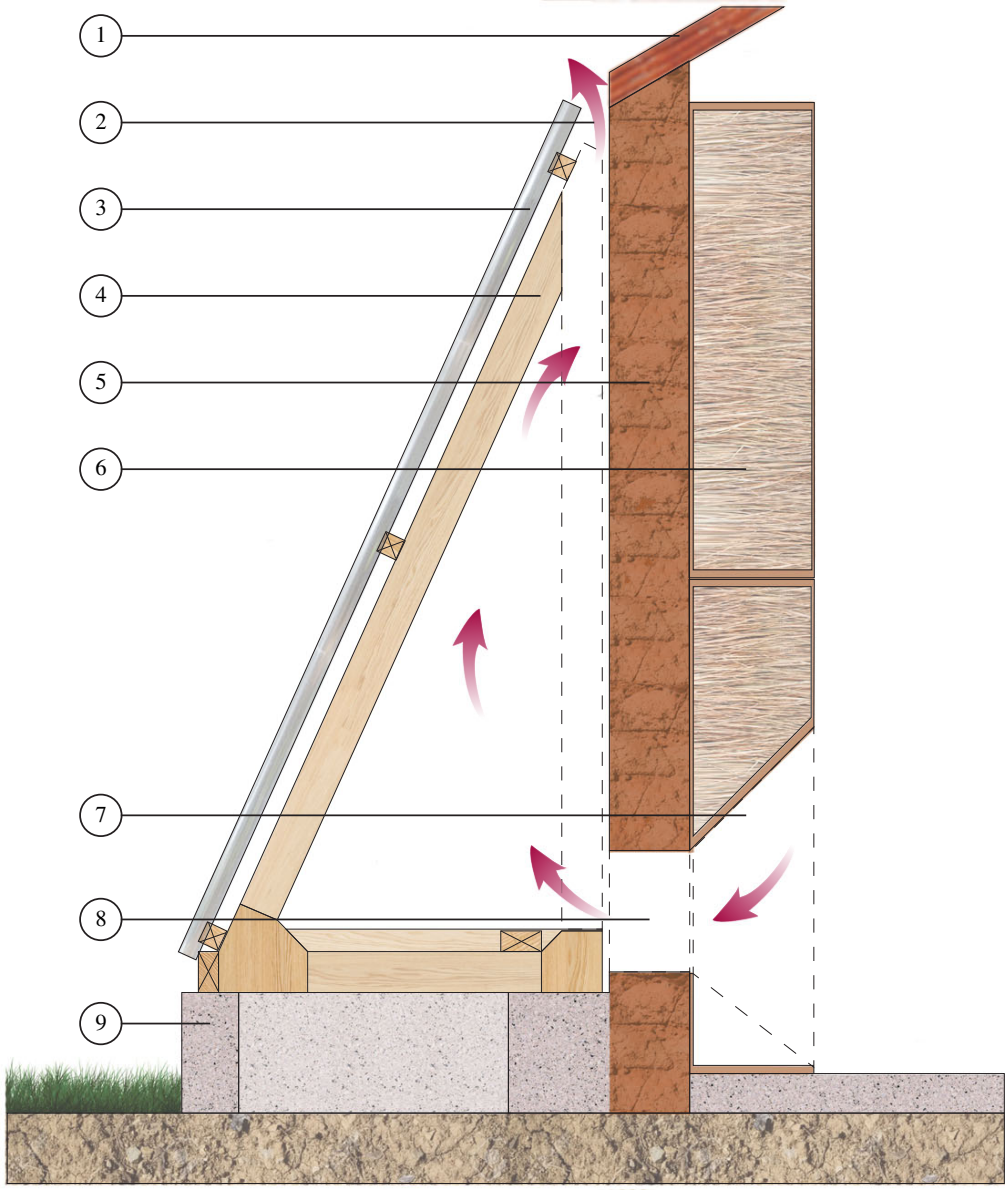
Air movement through the solar chimney. Where 1 = corrugated metal roof at wall level, 2 = hot air outlet, 3 = transparent corrugated sheet, 4 = wood frame, 5 = mud-brick wall (painted black on the outside), 6 = thatch inside plywood frames, 7 = plywood isolation frame, 8 = wall hole to allow air flow, 9 = concrete plinth, and red arrows show circulation of hot air. Dotted lines represent section elements that are not coloured for better understanding.

For single-roomed rural houses without electricity, we hypothesized that solar chimneys, a type of passive ventilation, would reduce indoor temperature. Briefly, a solar chimney is a device positioned on the sunny side of the house. The chimney, heated by direct sunlight, warms the air in the chimney, causing it to rise through an inlet in the bottom of the wall and out of the top of the chimney, drawing in cooler air from the room, increasing ventilation indoors and cooling the room (figure 1). Passive ventilation has been used for centuries in hot climates, including wind catcher towers in Iran [13], traditional Malay houses [14] and, in the natural world, termite mounds [15]. Today, with increasing global temperatures, passive ventilation strategies are gaining favour to create comfortable living and working environments without excessive energy consumption and expenditure [16,17]. In Venezuela, a solar chimney reduced indoor temperature at the hottest time of the day by 2–4°C, compared with outdoor temperature [18]. While, in Colombia, a solar chimney combined with a ground-air heat exchanger, a underground duct designed to move cooler air into the house after hot air has been expelled by the solar chimney, reduced indoor temperature by 1°C between 10.00 and 16.00 compared with the control house [19].

One alternative cooling strategy is to use active ventilation. For houses with electricity, bedrooms can be made cooler by using a fan that increases air flow across the body, helping to keep the body cool. Although ceiling fans are common in modern houses in the tropics and sub-tropics, studies on their effect on indoor temperature are scarce. In Singapore, office workers were more comfortable at 26°C with fan-assisted air movement compared with 23°C without fans [20]. Although there is anecdotal evidence that strong ceiling fans prevent mosquitoes flying, this has not, to our knowledge, been tested experimentally.

In the present study, we assessed whether passive and active ventilation would make a typical rural Gambian house more comfortable at night and reduce mosquito house entry, by lowering indoor CO_2_ concentrations, making it less likely that a malaria mosquito would enter a house. Constructing houses that are well ventilated and cooler could contribute to a reduction in malaria transmission by (i) increased human comfort indoors leading to earlier entry into the house in the evening (and avoidance of outdoor biting malaria mosquitoes) and increased use of ITNs, and (ii) reduction in indoor CO_2_ concentrations, reducing attraction of malaria mosquitoes to the house.

## 2. Methods

### 2.1. Study design

This was an experimental study using two identical single-roomed experimental houses, each occupied by two adults. We conducted two experiments comparing: (i) a house with a solar chimney and one without, and (ii) a house with a ceiling fan turned on and one with a fan turned off. Each experiment lasted 32 nights. In both experiments, house treatments were rotated every four nights. The initial allocation of treatment or control to houses was random. Sleeper pairs were rotated nightly between houses for the duration of the experiment.

### 2.2. Study area

The study was conducted in Wali Kunda field station (13° 34″25′ N, 14° 55″28′ W), located in the Central River Region, The Gambia. This is an area of flat Sudanese savannah, close to a large rice-cultivated area. The study took place in 2021 during the rainy season, from 23 July to 24 October, when the density of *Anopheles gambiae* *sensu lato*, the primary malaria vector, is the highest [3].

### 2.3. Experimental houses

**Figure 2.**
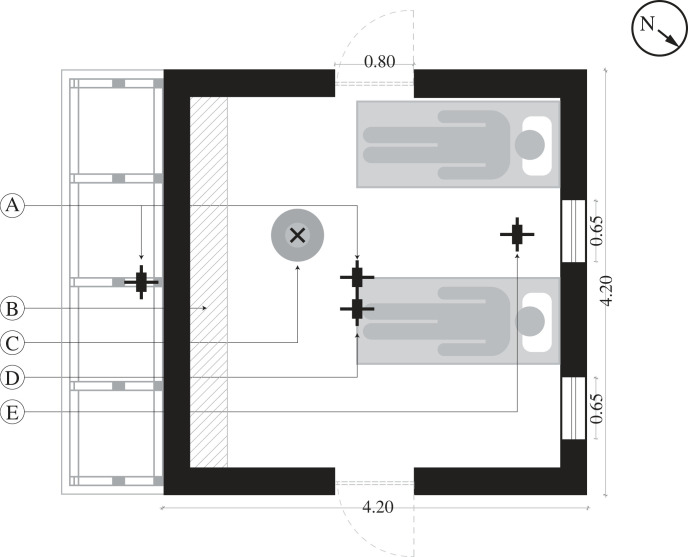
Position of solar chimney and data loggers in experimental houses. Where A = temperature loggers inside the solar chimney and centre of the house, B = insulated wall, C = light trap, D = evaporimeter and E = CO_2_ logger. Data loggers recording ambient temperature and CO_2_ were placed in a Stevenson screen between the two experimental houses (not shown).

Two experimental houses were used as described previously [7]. Briefly, both houses were the average size of a modern single-roomed house in rural Gambia and their construction reflected common practices in the country with a metal roof, closed eaves and two badly fitting doors. The two experimental houses were located 10.0 m apart and were 4.20 by 4.20 m in floor area, with walls 2.20 m high. They were constructed from sun-baked mud-blocks and corrugated metal roofs with no eave gaps. Each house had two 1.80 m high and 0.80 m wide doors located in the northeast and southwest facing walls of the house. Each door had narrow horizontal slits, each 20 × 800 mm, above and below the door, to simulate badly fitting doors, common in the region, and to allow mosquitoes to enter the building. The houses had two 0.65 m high and 0.65 m wide windows screened with PVC-coated fibreglass netting with a mesh size of 42 holes per cm^2^ (Vestergaard-Frandsen group, Kolding, Denmark) located on the northwest façade of the house at 1.20 m from the ground. Each house had two beds, located parallel to one another and with the head end of the bed closest to the wall with the screened windows (figure 2).

### 2.4. Interventions

#### 2.4.1. Solar chimney

**Figure 3.**
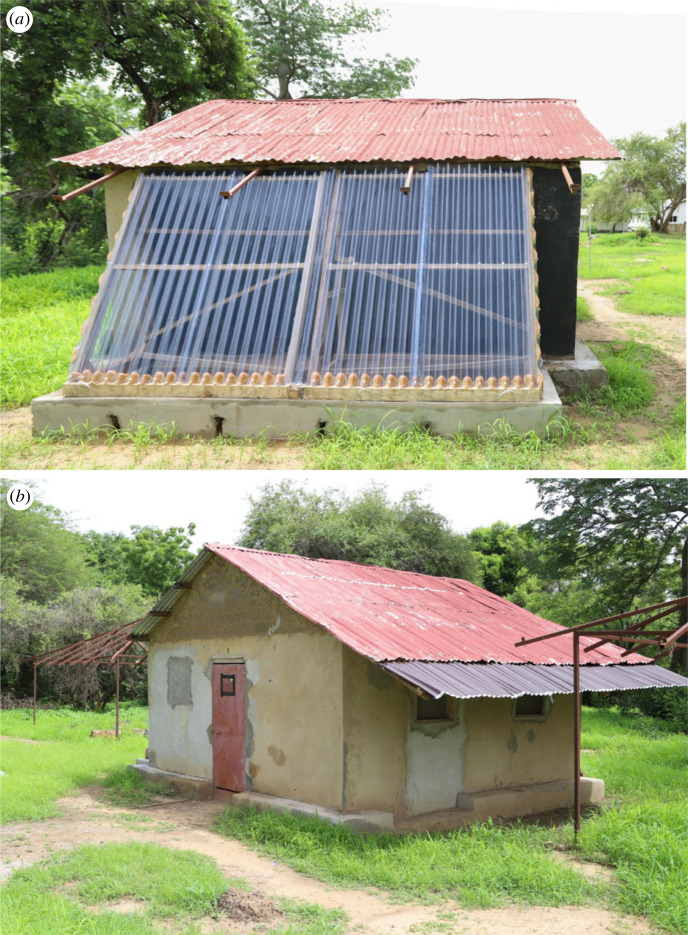
Experimental house with solar chimney on southeast facing wall (*a*) and extended roof on northwest elevation (*b*).

A panelled solar chimney was built on the southeast facing façade of the experimental houses to maximize solar radiation (figure 3). It was a lean-to structure made of two 2.15 m panels and two 0.80 m triangular lateral panels. Each module had a timber frame (2.15 × 1.70 m) supporting transparent corrugated polycarbonate panels (1.00 m × 2.40 m × 6 mm). Panels were fixed on a concrete base (0.30 m high and 0.30 m wide) which had four slits (0.08 by 0.04 m) on the longest side to prevent rainwater accumulating during a heavy downpour. The panels were sealed on the frame using expanding foam and silicone sealant (Transparent Acetci Silicone Sealant, INGCO, Ghana) to prevent leakage of hot air. At the top of the solar chimney there was a 30 mm gap between the panels and wall to allow hot air to leave the chimney. The wall on which the solar chimney was fixed was painted with matt black water-based paint (National Paints, Abu Dhabi, UAE) to increase heating within the solar chimney. When the house was acting as the control, we painted the wall using a wall-coloured (grey/brown) water-based paint (National Paints, Abu Dhabi, UAE) to restore the regular colour. There were four rectangular holes, made in the base of the wall of the house, each 0.19 × 0.37 m in area, 0.20 m above the floor. Each hole was made by removing a single mud-brick and replacing it with a 12 mm plywood frame to support the wall while the experiment was taking place. The holes were unscreened since we did not want to restrict air flow into the solar chimney and the holes were covered with pieces of 12 mm plywood when the house was acting as the control. On the internal face of the wall with the solar chimney we built a 0.30 m wide insulation layer made with eight plywood frames (0.95 by 1.17 m on top and 0.95 by 1.22 m at the bottom) filled with thatch to prevent the wall radiating heat into the room. The frames at the bottom of the wall had holes aligned with the holes in the house wall that allowed air movement from the room into the solar chimney (figure 1). As well as installing the solar chimney, we extended the metal roof on the northwest elevation by 0.80 m to shade the screened windows (figure 3). The roof extension panel was removed when the house was acting as control.

#### 2.4.2. Ceiling fan

One ceiling fan (F-56MZ2 56″ Ceiling Fan, 220 V, 14–70 W at 50 Hz, Panasonic, Japan) with 1.42 m blades and a diameter of 1.40 m was installed in each house and powered by 220 V mains electricity. Each fan was positioned in the centre of each room, anchored to an existing steel beam supporting the metal roof, 2 m above the floor. At 21.00 one of the fans was switched on at the highest speed of 268 revolutions per minute and switched off at 07.00 the next day. In the control house the fan was not connected to the power supply. Any power outages during the experiment were recorded.

### 2.5. Human subjects

The study was explained in a community meeting with male villagers in Mandinka, the local language. Four healthy adult men (greater than 18 years old) provided signed-witnessed consent and were hired to sleep four nights a week for the duration of the study. Women were excluded from the study due to cultural and religious reasons. Every night each pair of men slept, with their heads pointing northwest, under an ITN (Olyset Net, Sumitomo Chemicals, Japan; 1.3 m wide × 1.8 m long × 1.5 m high), from 21.00 to 07.00 the following morning. Two field assistants were posted outside the experimental huts throughout the study to assist the sleepers if they needed to briefly leave the house during the night and to ensure the men were sleeping under the ITNs. Each pair of sleepers were rotated each night so that at the end of the experiment each pair had slept 16 nights in each one of the experimental houses.

### 2.6. Entomology

Mosquitoes were collected indoors using CDC light traps (Centers for Disease Control and Prevention, Miniature light trap model 512, US John W. Hock Ltd, Gainesville, USA) operating from 21.00 to 07.00. After collection, any mosquitoes still alive were knocked down in a −20° C freezer and identified using standard morphological identification keys [21,22]. A random subsample of *An. gambiae* complex specimens were further identified using polymerase chain reaction (PCR) [23–25].

### 2.7. Environmental measurements

Indoor temperature and relative humidity were measured in each experimental house for the duration of the study every 30 min using a data logger (Tiny Tag, TGU 4500), positioned in the centre of the room, 1 m above the floor, and inside the solar chimney, 1 m above the floor of the house. Evaporation was measured from 21.00 to 07.00 with a Piche evaporimeter (Casella, Sycamore, USA) located 1.20 m from the floor in the middle of the room hanging from the roof structure. CO_2_ was recorded every 30 s from 21:00 to 07:00 nightly with a data logger (1% CO_2_ + Rh/T Data Logger GasLab, accurate to ±30 ppm ±3% of measured value) located between the beds, near the head of the bed, 1.2 m above the floor (figure 2).

Outdoor temperature and relative humidity were recorded every 30 min and CO_2_ every 30 s with a data logger (Tiny Tag, TGU 4500 and 1% CO_2_ + Rh/T Data Logger GasLab, accurate to ±30 ppm ±3% of measured value) installed in a Stevenson screen positioned midway between the two experimental houses. A Stevenson screen is a white louvred box approximately 1.2 m above the ground used to shelter data loggers from precipitation and direct heat radiation, while still allowing air to circulate freely around them. Outdoor wind speed and wind direction were recorded with an automatic weather station (MiniMet, Skye Instruments, Llandrindod Wells, UK) every 30 min, located northeast of the two houses, 10 m from each house.

### 2.8. Cost of intervention

The cost of materials for the two interventions were estimated, excluding labour and operating costs. Costs were extracted from study records and were recorded in the currency of expenditure (Great Britain Pounds, GBP or Gambian dalasi, GMD). Costs were converted to United States Dollar (USD) using the mean exchange rate for the study period (29 June 2021) of 1 GMD = 0.019571 USD and 1 GBP = 1.3844 USD. Materials purchased for the construction of the solar chimney were locally available in the capital city of Banjul and transported to Wali Kunda.

### 2.9. Statistical analysis

The primary outcome was the indoor density of *An. gambiae* s.l.. The sample size was estimated via simulation based on a previous experiment done in the same area in 2018, where the mean number of *An. gambiae* s.l. collected in metal-roofed houses with two windows and badly fitting solid and screened doors was 29.3 *An. gambiae* s.l./night (standard deviation = 20.1). To detect a 50% reduction in mosquitoes caught in the house with improved ventilation, at the 5% level of significance, with 80% power would require 31 nights of collection (rounded up to 32 nights). The same number of nights was also considered sufficient to show a significant difference in the other study outcomes. Temperature, relative humidity and CO_2_ concentration were analysed for two periods, from 21.00 to 23.30 (23.59 for CO_2_), the time most people go to bed and decide whether to use a bed net or not and from 00.00 to 07.00, when they are asleep [26].

Statistical analysis was performed using Stata version 15 (StataCorp., College Station, TX, USA). Mosquito collections are presented as means with 95% confidence intervals and analysed separately for each major taxon. We assessed the effect of house treatment on indoor climate and mosquito house entry using generalized linear modelling, using a negative binomial model with a log link function for mosquito count data, while comparisons of temperature, relative humidity, evaporation and CO_2_ were made using linear regression. In addition to house treatment, we included house position, sleeper pair and night in the models as fixed effects. We calculated protective efficacy (1 − mean ratio × 100) of each intervention to reduce mosquito house entry. Evaporation was calculated nightly for each house by subtracting the level of water recorded at 07.00 from that recorded at 21.00.

We used polar plots to depict the direction and strength of the wind during the day and night.

Computational fluid dynamics (CFD) modelling was used to simulate CO_2_ concentrations and indoor temperature distribution using Ansys Fluent (v. 19). Model assumptions and set-up configurations were as follows: (i) the house was based on the structure of an experimental house and was assumed air-tight, except for gaps at the top and bottom of the doors, and the screened windows, (ii) two men were modelled as geometrically simplified mannequins with rectilinear body shapes, (iii) exhalation velocity of the mannequins was 0.77 m s^−1^ upwards, with 40 000 ppm of CO_2_, (iv) the temperature of exhaled breath was 33°C and body temperature 36°C, (v) background CO_2_ was 555 ppm, and outdoor air temperature 25°C (based on data collected in this study, table 2), (vi) since the outdoor night wind speed in the study site was measured by the weather station as close to zero most of the time, a wind speed of 0.1 m s^−1^ was applied (vii) bed nets and screened windows were assumed to attenuate air flow by 64% [4], (viii) air is incompressible, and air flow is steady turbulent flow, (ix) we used realizable k – ɛ turbulent models with scalable wall functions since they best matched field data, and (x) the entire model had 4.1 million polyhedral cells, with 1 mm cells for the mouths and 10 mm for the nets. CFD simulations were verified against: (i) detailed field data collected in Tanzania during the rainy season [10], and (ii) CO_2_ and temperature data logger recordings made in this study. The average indoor temperature and CO_2_ distributions inside the houses during sleeping hours were studied based on the measurements at 02.00 and the data logger (probe) position was assumed to be in the centre of the house 1 m above the ground.

**Table 2.** Average outdoor and indoor temperature and CO_2_ concentrations in houses with and without a solar chimney, and with and without operating ceiling fans. Generalized linear modelling results, adjusted for house position, sleeper pair and night. CI = confidence intervals. In both experiments CO_2_ was not recorded between 07.00 and 20.59 as the houses were unoccupied. Temperature was not recorded during the day in the ceiling fan experiment since the houses were unoccupied and the ceiling fan was not operational at this time.

temperature									
house typology	21.00 to 23.30			00.00 to 07.00			07.30 to 20.30		
	unadjusted mean temperature°C (95% CI)	coefficient (95% CI)	*p* value	unadjusted mean temperature°C (95% CI)	coefficient (95% CI)	*p* value	unadjusted mean temperature°C (95% CI)	coefficient (95% CI)	*p* value
*solar chimney experiment*									
outdoors	26.1 (25.5–26.8)	—		24.9 (24.5–25.4)	—		31.6 (30.9–32.3)	—	
control	29.5 (28.9–30.2)	—		28.1 (27.6–28.6)	—		30.4 (29.8–30.9)	—	
chimney	29.6 (29.0–30.2)	0.03 (−0.08–0.14)	0.64	28.1 (27.6–28.6)	−0.01 (−0.11–0.09)	0.85	30.5 (30.0–31.1)	0.19 (0.08–0.30)	0.001
*ceiling fan experiment*									
outdoors	25.6 (25.2–26.1)	—	—	24.4 (24.0–24.7)	—	—			
control (fan off)	29.7 (29.3–30.1)	—	—	28.0 (27.7–28.3)	—	—			
fan on	29.6 (29.2–30.0)	−0.08 (−0.17–0.01)	0.09	27.9 (27.5–28.2)	−0.06 (−0.13–0.01)	0.08			
CO_2_ concentrations									
house typology	21.00 to 23.59			00.00 to 07.00					
	unadjusted mean CO_2_ (ppm) (95% CI)	coefficient (95% CI)	*p* value	unadjusted mean CO_2_ (ppm) (95% CI)	coefficient (95% CI)	*p value*			
*solar chimney experiment*									
outdoors	555 (517–593)	—		547 (518–576)	—				
control	690 (653–726)	—		686 (648–723)	—				
chimney	667 (627–707)	−20.39 (−58.09–17.31)	0.29	645 (616–674)	−38.90 (−68.07 – −9.73)	0.009			
*ceiling fan experiment*									
outdoors	648 (613–682)	—	—	624 (594–654)	—	—			
control (fan off)	793 (761–826)	—	—	721 (687–755)	—	—			
fan on	752 (729–775)	−41.5 (−79.8 - −3.15	0.03	698 (675–721)	−31.7 (−69.0–5.72)	0.1			

Human comfort was assessed using the software package Ladybug (Ladybug Products, Athol, ID, USA), which was used to estimate the proportion of time occupants of various house typologies spent in the ‘comfort zone’. The comfort zone is defined by the comfort polygon for temperature and relative humidity and provides an estimated proportion of people satisfied with the indoor climatic comfort. We assumed that from 21.00 to 23.59 men were seated and quiet and wore trousers, briefs and T-shirts. From 00.00 to 07.00 the men were sleeping. Human comfort analysis for the ceiling fan experiment assumed an air velocity (derived from CFD modelling) of 0.36 m s^−1^, while air velocity was assumed to be 0.0 m s^−1^ in control houses and the solar chimney house. For each house treatment, we calculated the proportion of time the indoor climate was in the comfort zone for two periods: 21.00 to 23.59, when people retire to bed, and 00:00 to 07:00, when people are usually sleeping [26].

## 3. Results

### 3.1. Solar chimney (passive ventilation)

#### 3.1.1. Entomology

A total of 2558 female mosquitoes were collected in the light traps during the study, of which 10% (246/2558) were *An. gambiae* s.l., 88% (2239/2558) *Mansonia* spp., 1.8% (47/2558) *Culex* spp. and the rest were other anophelines and *Aedes aegypti* (electronic supplementary material S1, table S2). Female *An. gambiae* s.l. specimens identified using PCR comprised *An. coluzzii* (67%, 20/30), *An. arabiensis* (23%, 7/30) and inconclusive (10%, 3/30) in the house with the solar chimney installed, and as *An. coluzzii* (73%, 22/30) and *An. arabiensis* (27%, 8/30) in the control house.

**Figure 4.**
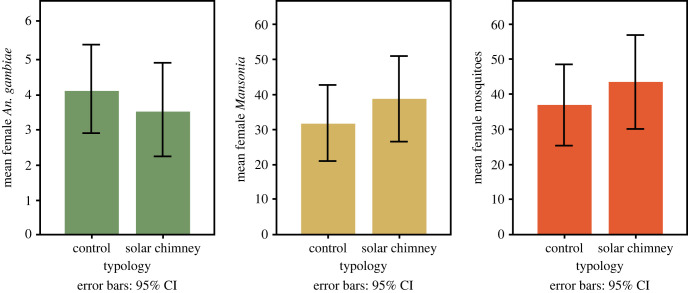
Mean mosquito numbers per night recorded for solar chimney experiment. For female *An. gambiae* s.l., female *Mansonia* spp. and all female mosquitoes (*Anopheles* spp., *Mansonia* spp., *Culex* spp. and *Aedes* spp.).

Unadjusted analysis showed mean nightly female *An. gambiae* s.l. numbers of 3.6 (95% CI 2.2 to 4.9) in the solar chimney house and of 4.1 (95% CI 2.8 to 5.4) in the control house (figure 4). For *Mansonia* spp. the mean number in the solar chimney house was 38.5 (95% CI 26.3 to 50.7) and 31.5 (95% CI 20.9 to 42.1) in the control house per night. There was a nightly average of 43.3 (95% CI 30.2 to 56.5) female mosquitoes in the solar chimney house and 36.6 (95% CI 25.4 to 47.8) female mosquitoes in the control house.

Analysis adjusted for confounders (house number, sleeper pair and night) did not identify a significant difference between mosquito numbers caught in the house with the solar chimney compared with the control house (*An. gambiae* s.l.: protective efficacy (PE) = 28%, 95% CI = −39%–63%, *p* = 0.32; *Mansonia* spp.: PE = −30%, 95% CI = −122%–23%, *p* = 0.33; all female mosquitoes: PE = −22%, 95% CI = −106%–28%, *p* = 0.46; table 1).

**Table 1.** Female mosquitoes collected from houses with and without a solar chimney and with and without operating ceiling fans. Generalized linear modelling results, adjusted for house position, sleeper pair and night. CI = confidence intervals.

house typology	total number of mosquitoes	mean/night (95% CI)	mean ratio (95% CI)	protective efficacy (95% CI)	*p* value
female *An. gambiae* s.l.					
*solar chimney experiment*					
control	132	4.1 (2.8–5.4)	—		
chimney	114	3.6 (2.2–4.9)	0.72 (0.37–1.39)	28% (−39%–63%)	0.32
*ceiling fan experiment*					
fan off (control)	101	3.5 (2.1–4.9)	-		
fan on	37	1.1 (0.3–1.8)	0.09 (0.03–0.28)	91% (72% - 97%)	<0.001
*Mansonia* spp.					
*solar chimney experiment*					
control	1008	31.5 (20.9–42.1)	—		
chimney	1231	38.5 (26.3–50.7)	1.30 (0.77–2.22)	−30% (−122%–23%)	0.33
*ceiling fan experiment*					
fan off (control)	2491	85.9 (56.0–115.8)	—		
fan on	978	27.9 (14.9–41.0)	0.29 (0.16–0.52)	71% (48% - 84%)	<0.001
all female mosquitoes (*Anopheles* spp., *Mansonia* spp., *Culex* spp. and *Aedes* spp.)					
*solar chimney experiment*					
control	1171	36.6 (25.4–47.8)	—		
chimney	1387	43.3 (30.2–56.5)	1.22 (0.72–2.06)	−22% (−106%–28%)	0.46
*ceiling fan experiment*					
fan off (control)	2638	91.0 (60.3–121.6)	—	—	
fan on	1032	29.5 (15.7–43.3)	0.28 (0.15–0.49)	72% (51%–85%)	<0.001

#### 3.1.2. Environmental measurements

**Figure 5.**
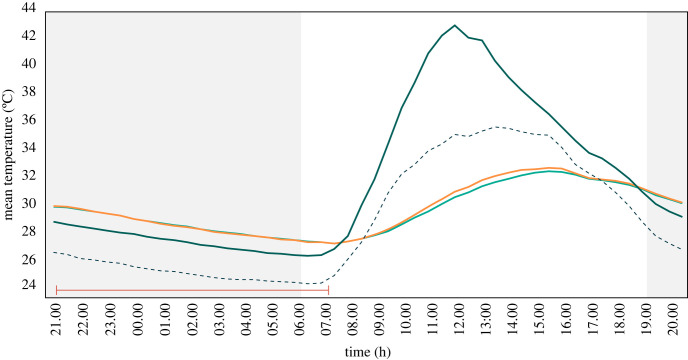
Indoor and outdoor temperatures recorded during the solar chimney experiment. Where mean outdoor temperature = dotted line, indoor temperature in control hut = light green line, indoor temperature in solar chimney house = orange line, temperature in solar chimney = dark green line, night = grey section and duration of experimental night = red line.

Between 07.00 and 20.30 the average temperature was 30.5°C in the house with the solar chimney and 30.4°C in the control house, 1.1°C and 1.2°C lower than outdoor levels (figure 5, table 2). During the day, the chimney had a mean temperature of 35.0°C, 4.6°C higher than the mean indoor temperature of both houses and 3.4°C higher than the outside average for the same period. Around 18.30 the chimney stopped being warmer than the adjoining house, a trend that was maintained until around 07.30. Between 21.00 and 23.30, the unadjusted average temperature was 29.6°C inside the house with the solar chimney and 29.5°C inside the control house, 3.5°C and 3.4°C higher compared with outdoor levels (figure 5). The mean temperature inside the solar chimney from 21.00 to 23.30 was 28.4°C, 1.1°C lower than the adjoining house but 2.3°C higher than outdoor levels. During the second part of the night between 00.00 and 07.00 the average temperature in both houses was 28.1°C, 3.2°C higher than outdoors. At this time, the unadjusted average temperature inside the chimney was 27.1°C, 1.0°C lower than inside the houses and 2.2°C higher than outdoors.

Mean indoor temperature in the house with the solar chimney was similar to the control house during the night (table 2). During the day, however, the house with a solar chimney was slightly hotter than the control house. Relative humidity levels were similar between house types (electronic supplementary material S1, table S1).

Indoor evaporation was higher at night (21.00 to 07.00) in the house with a solar chimney (mean 1.9 ml, 95% CI 1.5 to 2.2) compared with the control house (mean 1.4 ml, 95% CI 1.0 to 1.8), although the result was non-significant (*p* = 0.08) (table 3).

**Table 3.** Indoor evaporation during both experiments. Linear regression adjusted for house position, sleeper pair and night. CI = confidence intervals.

house typology		mean / night (95% CI)	coefficient (95% CI)	*p* value
solar chimney	control	1.4 (1.0–1.8)	—	—
	intervention	1.9 (1.5–2.2)	0.48 (−0.06–1.03)	0.08
ceiling fan	control	1.3 (0.9–1.7)	—	—
	intervention	2.6 (2.2–3.0)	1.43 (0.85–2.01)	<0.001

Wind was predominantly from the northwest during the first part of the night (21.00 to 23.59) and from the northwest and southeast during the second part of the night (00.00 to 07.00). Mean wind speed was 0.10 m s^−1^ (95% CIs 0.07 to 0.12) from 20.00 to 23.59 and 0.07 m s^−1^ (95% CIs 0.6 to 0.8) from 00.00 to 06.59 (electronic supplementary material S1, figure S2).

**Figure 6.**
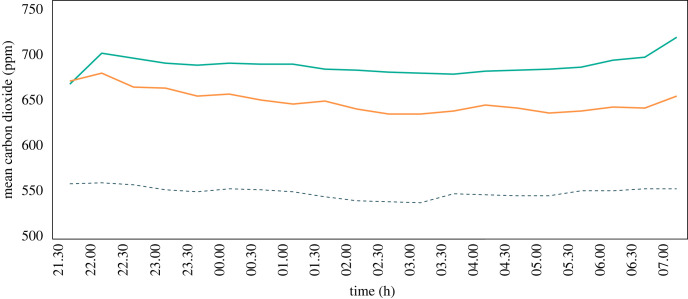
Average indoor and outdoor night-time CO_2_ concentrations recorded during the solar chimney experiment. Where outdoor levels = dotted line, control house = turquoise line and house with solar chimney = orange line.

CO_2_ levels increased immediately after the sleepers entered the experimental houses peaking at around 22.00. Thereafter, CO_2_ levels slowly declined before rising sharply after 06.30 when the sleepers woke (figure 6). CO_2_ levels were consistently higher inside than outside throughout the night. There was a lower CO_2_ concentration in a house with a solar chimney than a house without a solar chimney in both parts of the night, although the result reached statistical significance only after midnight (table 2). From 21.00 to 23.59 CO_2_ levels were 667 ppm in the house fitted with the chimney and 690 ppm in the control house (*p* = 0.29), while outside it was 555 ppm. From 00.00 to 07.00 CO_2_ levels were 645 ppm in the house fitted with the chimney and 686 ppm in the control house (*p* = 0.009), while outside it was 547 ppm. Across the entire night (21.00 to 07.00), CO_2_ levels were significantly lower in the house fitted with the chimney (653 ppm) than the control house (683 ppm; *p* < 0.001).

#### 3.1.3. Human comfort

**Figure 7.**
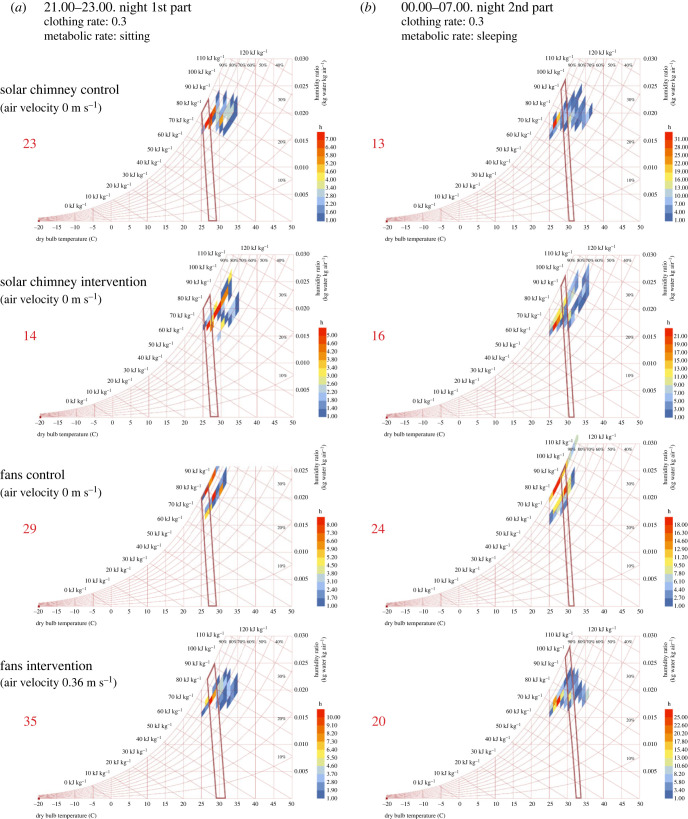
Psychrometric charts showing the human comfort index of adults in houses with and without a solar chimney, and with and without a ceiling fan. (*a*) Readings, shown as coloured polygons, made from 21.00 to 23.59. (*b*) Readings made from 00.00–06.00. Human comfort analysis for the ceiling fan assumes an air velocity experienced by the sleeper of 0.36 and 0.0 m s^−1^ for the solar chimney house and control houses. Each data point represents a combination of temperature and relative humidity (and air velocity for ceiling fan) at different times of the night. Data points that fall within the black polygons represent values that are known to be comfortable for lightly dressed adults sitting or sleeping. Data to the left of the polygon represents values that will be perceived as too cold while data to the right will be considered too hot. Values in red indicate the percentage of readings deemed to be comfortable.

During the first part of the night, psychrometric analysis indicated that the solar chimney house was less comfortable than the control house, with a human comfort index of 14% in the solar chimney house compared with 23% in the control house (figure 7). During the second part of the night, both the control and solar chimney house were uncomfortable, with a human comfort index of 16% in the solar chimney house and 13% in the control house.

#### 3.1.4. Computational fluid dynamics simulations

**Figure 8.**
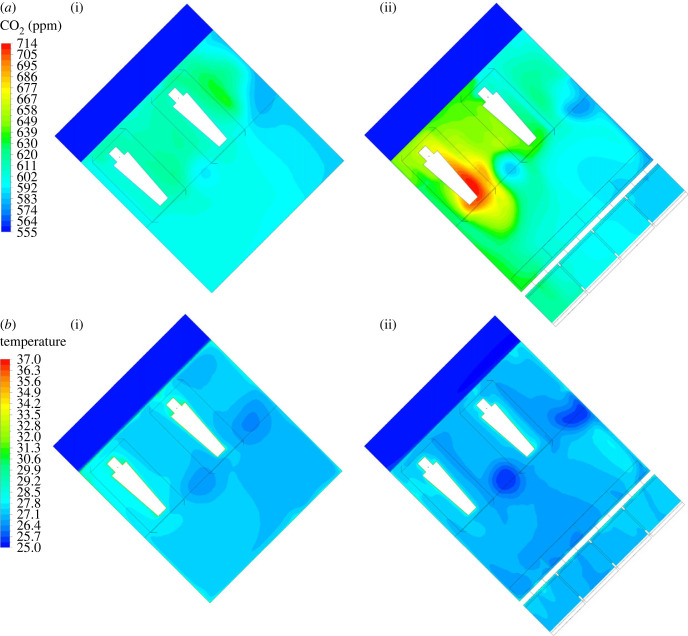
CFD simulations of (*a*) CO_2_ distribution and (*b*) indoor temperature in the house without (i) and with (ii) solar chimney. The solar chimney is depicted in (ii) as an additional rectangular shape adjoining the house. CO_2_ distributions shown are at the height of the two sleeping adults.

Simulated temperature and CO_2_ at the probe locations closely matched the field data. The simulated indoor temperatures of the two houses at the probe location were similar at 27.6°C in the house with solar chimney and 27.3°C in the control house (figure 8). The average indoor temperature at the vertical height of the sleepers was 27.0°C in the house with solar chimney and 27.4°C in the control house. The indoor CO_2_ distributions were simulated with around 10% average discrepancy against the field data at the probe location. There was a negligible difference in the simulated CO_2_ concentration between the two houses at 627 ppm in the solar chimney house and 620 ppm in the control house. The simulation results show that the CO_2_ concentration around the sleeper near a door in the house with the solar chimney was higher by about 100 ppm than the control house.

#### 3.1.5. Cost of intervention

Materials for construction of the solar chimney cost 517.2 USD.

### 3.2. Ceiling fans (active ventilation)

#### 3.2.1. Entomology

A total of 3670 female mosquitoes were collected during the ceiling fan study, of which, 3.8% (138/3670) were *An. gambiae* s.l., 94.5% (3469/3670) *Mansonia* spp., 1.1% (42/3670) *Culex* spp. and the rest were other anophelines and *Ae. aegypti* (electronic supplementary material S1, table S1). Specimens identified as female *An. gambiae* s.l. were identified by PCR analysis as *An. coluzzii* (83%, 25/30), *An. arabiensis* (10%, 3/30) and *An. gambiae* s.s./*coluzzii* (3%, 1/30) and inconclusive (3%, 1/30) in the house with the working fan, and as *An. coluzzii* (80%, 24/30), *An. arabiensis* (17%, 5/30) and *An. gambiae* s.s./*coluzzii* hybrids (3%, 1/30) in the house with the fan off.

**Figure 9.**
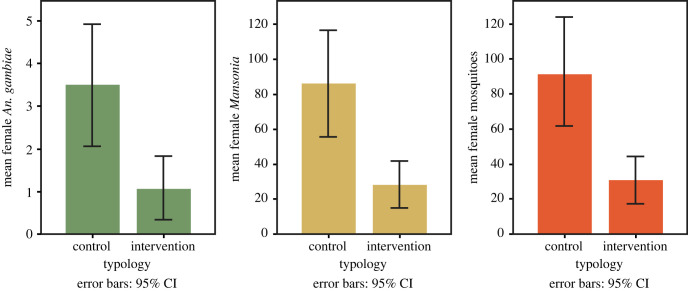
Mean mosquito numbers per night recorded for ceiling fans experiment. For female *An. gambiae* s.l., female *Mansonia* spp. and all female mosquitoes (*Anopheles* spp., *Mansonia* spp., *Culex* spp. and *Aedes* spp.).

Unadjusted analysis of the mean number of female *An. gambiae* s.l., *Mansonia* spp. and female mosquitoes showed that there were fewer mosquitoes in the house with the ceiling fan than in the control house (figure 9). The mean number of female *An. gambiae* s.l. in the house with the working fan was 1.1 (95% CI 0.3–1.8) and 3.5 (95% CI 2.1–4.9) in the control house (table 1). Mean numbers of *Mansonia* spp. in the house with working fan was 27.9 (95% CI 14.9–41.0) and 85.9 (95% CI 56.0–115.8) in the control house. The mean numbers of total female mosquitoes in the house with a working fan was 29.5 (95% CI 15.7–43.3) and 91.0 (95% CI 60.3–121.6) in the control house.

Analysis adjusted for confounders (house number, sleeper pair and night) showed a 91% (95% CI 72%–97%, *p* < 0.001)) reduction in *An. gambiae* s.l. and 71% (95% CI 48%–84%, *p* < 0.001) reduction in *Mansonia* spp. in houses with operating fans compared with the control house (table 1). Similar reductions were seen with all female mosquitoes combined.

#### 3.2.2. Environmental measurements

**Figure 10.**
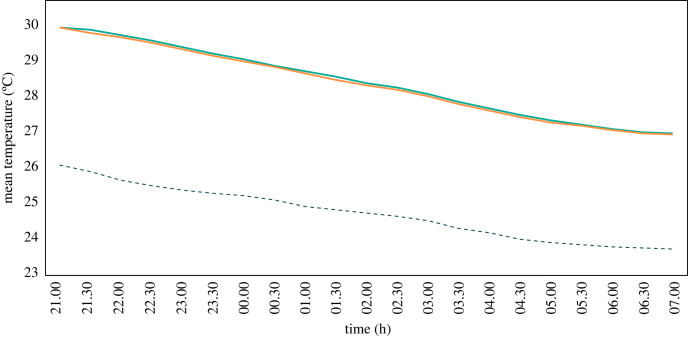
Average indoor and outdoor temperatures recorded during experimental night of the ceiling fans experiment. Outside (dotted line), control hut (turquoise line) and intervention hut (orange line).

The average temperature between 21.00 and 23.30 was 29.6°C inside the house with the fan and 29.7°C inside the control house, around 4.0°C higher than outdoor levels. Between 00.00 and 07.00, the mean temperature was 27.9°C in the house with the fan and 28.0°C in the control house, around 3.5°C higher than outdoor levels (figure 10). There was no significant difference in temperature after adjusting for house position, sleeper pair and night (table 2). Relative humidity was also similar between the two houses (electronic supplementary material S1, table S3).

Evaporimeter measurements showed the water volume decreased by 2.6 ml (95% CI 2.2–3.0) in the house with a working fan compared with 1.3 ml (95% CI 0.9–1.7) in the control house (*p* < 0.001) (table 3).

Wind was predominantly from the north during both parts of the night (21.00 to 07.00) and mean wind speed was 0.03 m s^−1^ (95% CIs 0.01–0.04) from 20.00 to 23.59 and 0.07 m s^−1^ (95% CIs 0.05 to 0.08) from 00.00 to 06.59 (electronic supplementary material S1, figure S3).

**Figure 11.**
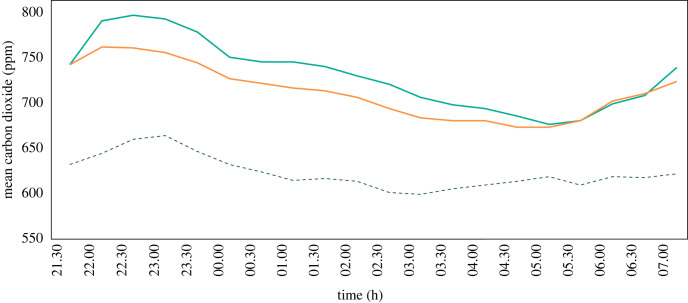
Average indoor and outdoor night-time CO_2_ concentrations recorded during the ceiling fan experiment. Outside (dotted line), house with fan turned off (turquoise line) and house with fan turned on (orange line).

During the ceiling fan experiment, CO_2_ levels peaked around 22.00, shortly after the sleepers entered the house (figure 11). Thereafter, CO_2_ levels declined until 05:30, when there was an increase. This same pattern was observed in both the intervention and control house. During the night CO_2_ concentrations were substantially higher indoors than outdoors. Indoor CO_2_ concentration at night was lower in the house with the ceiling fan than in the control house. During the first part of the night, the house with the fan had significantly lower CO_2_ levels at 752 ppm (95% CI = 729–775) compared with the control house at 793 ppm (95% CI = 761–826, *p* = 0.03), while the outdoor CO_2_ concentration was 648 ppm (table 2). During the second part of the night, CO_2_ levels were also lower in the house with the fan (698 ppm, 95% CI = 675–721) than in the control house (721 ppm, 95% CI = 687–755), although the difference was non-significant (*p* = 0.1). The outdoor CO_2_ concentration during the second part of the night was 624 ppm. Over the whole night, CO_2_ levels were lower in the house with the fan (713 ppm) than in the control house (736 ppm, *p* < 0.001).

#### 3.2.3. Human comfort

During the first part of the night, the human comfort index reached 35% in the house with the ceiling fan but fell to 20% during the second part of the night when it became too cold (figure 7). The human comfort index was similar in the control house during the first and second parts of the night, at 29% and 24%, respectively.

#### 3.2.4. Computational fluid dynamics simulations

**Figure 12.**
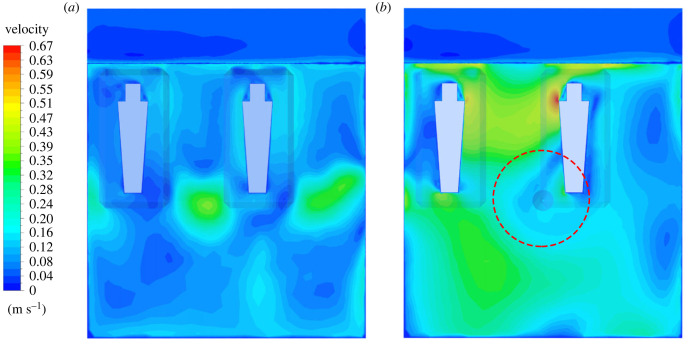
CFD simulations of air flow in the experimental house with no ceiling fan (*a*) and with the ceiling fan operational (*b*). The ceiling fan of diameter 1.40 m is depicted in (*b*) with a red dashed circle. CO_2_ distributions shown are at the height of the two sleeping adults.

The simulations show the fan's effect on circulating indoor air with a maximum downward speed of 2.69 m s^−1^. However, the air velocity drops as the flow hits the bed nets, resulting in the reduced air speed that eventually reaches the sleepers (electronic supplementary material S1, figure S4). The average air velocity around the sleepers (under the bed net) in the house fitted with the ceiling fan was around 0.36 m s^−1^ (maximum 0.67 m s^−1^), while it was 0.16 m s^−1^ (maximum 0.38 ms^−1^) in the control house. In respect of thermal condition, temperature at the probe position was 28.9°C with the ceiling fan operational which was higher than in the control house at 27.3°C (figure 12) according to the simulations. CO_2_ concentration at the probe position was 655 ppm in the house with the ceiling fan operational and 713 ppm in the control house.

#### 3.2.5. Cost of intervention

The ceiling fan cost 22.1 USD.

## 4. Discussion

Our pilot study established how passive and active ventilation affects indoor mosquito density, temperature, humidity, evaporation, CO_2_ concentrations and human comfort in modern single-roomed rural Gambian houses with metal roofs, closed eaves and badly fitting doors. The prototype solar chimney did not affect indoor mosquito density, temperature, humidity or evaporation. The almost identical indoor temperatures are probably due to two reasons: (i) while the solar chimney was hotter than the adjoining room during the day, the decline in temperature in the solar chimney after sunset was faster than indoors, resulting in no air movement across the room at night, (ii) the air flow through the windows is close to zero, owing to the minimal outdoor wind during sleeping hours (less than 0.1 m s^−1^). Although we measured a small (30 ppm) reduction in the CO_2_ concentration in the solar chimney house compared with the control house this was within the bounds of accuracy of the data logger (±30 ppm ±3% of measured value), and CFD modelling showed a negligible difference of around 10 ppm. Since CO_2_ is a major mosquito attractant [11], the lack of difference in CO_2_ concentration between the solar chimney house and the control house probably explains the lack of impact on mosquito density. CFD simulation also indicates that there was a 100 ppm higher CO_2_ concentration close to the sleeper near the door in the solar chimney house than the control house. The reason for this is unclear since air flow can be affected by multiple parameters.

The design of the solar chimney can be improved by altering the orientation of a house or the chimney, clearing obstructions around the house and using different construction materials. For example, orienting the house and chimney to receive more direct sunlight and removing shade trees will increase the temperature within the chimney. The solar chimney was built with lightweight material of low thermal mass, whereas a similar structure made from thick glass and higher thermal mass materials may have retained the heat for longer enabling the chimney to operate after sunset. Even though we painted the outside wall matt black to increase solar heating and added an insulation layer indoors to prevent heat transfer from the external face into the room, the substantial heating of the indoor air caused by the metal roof nullified this effect. Painting the roof white [27], adding a green roof (roof of a building that is partially or completely covered with vegetation and a growing medium, planted over a waterproofing membrane) or adding a ceiling could increase the cooling effect of solar chimneys by reducing the temperature of the room during the day.

Ceiling fans reduced indoor density of *An. gambiae* s.l., by 91%. Although there was no difference in indoor nightly temperature or relative humidity, the evaporation rate in the house with the ceiling fan was double that in the control house. The increase in evaporation was most likely due to the high indoor air velocity caused by the rotating fan. The average nightly CO_2_ concentration was 23 ppm lower in the house with the ceiling fan compared with the control house, although again this difference was probably within the bounds of accuracy of the data logger. The air velocity near the fan was 2.69 m s^−1^ while it was 0.36 m s^−1^ near the sleeper due to attenuation of air flow by the bed net [4]. Published literature suggests that high air velocity disrupts normal flight and host-seeking behaviour and it is probable that the higher air velocity in the house with the ceiling fan was the main factor contributing to the decreased indoor vector density. A field experiment in which mosquitoes approaching a host were exposed to different air velocities found that at air velocities above 0.8 m s^−1^ mosquito catches begin to drop off, with no mosquito flight observed when air velocity was 2.0 m s^−1^ [28]. In the laboratory, mosquitoes fly at a speed of 0.25 m s^−1^ when following an odour plume [29], suggesting a higher air velocity would restrict host location. Ceiling fans are also likely to disrupt the normal host-seeking behaviour of *An. gambiae* which specifically target the roof of bed nets due to the rising odour plume [30–32].

Night-time outdoor CO_2_ levels of around 550 ppm in the solar chimney experiment and 650 ppm in the ceiling fan experiment are high, reflecting the increased respiration from the soil and vegetation during the rainy season, where measurements are made close to the ground in a situation where there is low turbulent air.

Unexpectedly, the CFD modelled temperature at the probe position was higher in the house with the ceiling fan compared with the control house. It may be that hot air normally accumulating under the roof of the house was pressed downwards towards the sleeper. While ceiling fans improved human comfort in the first part of the night, they were considered too cool in the second half of the night when ambient temperature dropped. In practice, being too cold is not problematic since sleepers can be easily covered with a sheet or blanket. Keeping house inhabitants cool is more difficult.

CFD simulations allow us to model indoor climate and air flow *in silico* and support a more detailed understanding of air flow in the house than would be possible using data loggers positioned at fixed points in the house. For example, CFD simulation allowed us to understand the air velocity and movement under the fan including how the hot air was being pressed down and how the air velocity experienced by the sleeper was attenuated by the bed net. CFD simulation was used both during the design of the solar chimney and after the conduct of the experimental study when the model was parametrized with field data. The earlier simulations included construction details, which were different to what was eventually constructed on site. In addition, the physical properties of the construction materials had to be estimated, introducing further difficulties to match the real conditions. After the experimental study, the CFD models were reconstructed to better match the site conditions such as total number, and the dimensions of the openings in the solar chimney wall, ambient temperature and geometrical specifications of the thatch-filled insulation boxes.

There were several limitations to our study. Firstly, these are pilot studies using only two experimental houses. Secondly, two adults slept in a single-roomed house, while the median density of people in such houses in The Gambia is four adults and children (M Pinder 2022, personal communication). Thirdly, some rural houses in the Gambia are line houses, where single rooms are split in two by a dividing wall, where the dynamics of heating and CO_2_ may differ from this experimental set-up. Finally, we did not assess the impact of the water-based paint on mosquito attraction. We do not think this would have impacted the results because the paint was odourless and mosquito entry into the control and intervention house would have been impacted similarly.

Using active and passive methods to increase ventilation and indoor comfort levels in the tropics has been studied previously [33–35]. There are, however, to our knowledge, no experiments using solar chimneys to reduce mosquito numbers and keep the house cool at night. There have been several studies reporting the use of fans for reducing mosquito biting, although these were anecdotal, and for cooling indoors to increase bed-net use. In Kolkata, India, an observational study found that 53% of respondents reported not using a bed net and 80% used fans instead to avoid mosquitoes [36]. In Ghana, a trial of a small fan placed inside a bed net did not increase bed-net use, although the study did not have sufficient statistical power to detect a difference in bed-net use between the study arms [37].

While ceiling fans reduced mosquito entry and increased human comfort, there is still a pressing need to develop natural ventilation strategies because those at highest risk of malaria and heat stress do not have access to electricity. Air conditioning is not a good solution since it contributes to greenhouse gas emissions, which drive climate change and increase the risk of future extreme heat events. The 7% increase in renewable energy deployment, particularly solar power, between 2010 and 2020 in Africa [38] and improvements in battery storage could lend itself to battery-powered ceiling fans. The solar chimney was substantially more expensive than the ceiling fan at 517.2 USD compared with 22.1 USD, although the solar chimney was a prototype design needing further refinement and would benefit from economies of scale. The community acceptability of passive ventilation methods such as the solar chimney would also need to be explored.

## 5. Conclusion

The passive ventilation design tested was not able to reduce indoor temperature or mosquito density. Nevertheless, active ventilation using ceiling fans increases indoor air velocity, resulting in fewer mosquitoes entering these houses. Whether an optimized passive ventilation design would be able to achieve similar air velocities would need to be evaluated. Besides reducing mosquito ingress, improved house ventilation may increase bed-net use and thus protection from malaria mosquitoes. Changes to indoor ventilation could reduce indoor malaria transmission and maintain the gains in countries that have achieved elimination.

## Data Availability

The datasets used for the study are available as electronic supplementary materials (electronic supplementary material S2 – Entomological dataset and electronic supplementary material S3 – Environmental dataset) [39].
